# Cloning and characterization of an acidic lipase from a lipolytic bacterium in tempeh

**DOI:** 10.1186/s43141-023-00611-9

**Published:** 2023-12-01

**Authors:** Naswandi Nur, Antonius Suwanto, Anja Meryandini, Maggy Thenawidjaja Suhartono, Esti Puspitasari, Hyung Kwoun Kim

**Affiliations:** 1 Research Center for Applied Microbiology, National Research and Innovation Agency, Jl Raya Jakarta-Bogor KM 46, Cibinong, Bogor, 16911 West Java Indonesia; 2 Department of Biology, Faculty of Mathematics and Natural Science, IPB University, Bogor, 16680 Indonesia; 3 Department of Food Science and Technology, Faculty of Agricultural Engineering and Technology, IPB University, Bogor, 16680 Indonesia; 4 Department of Biotechnology Research and Development, PT Wilmar Benih Indonesia, Bekasi, 17530 Indonesia; 5 Division of Biotechnology, The Catholic University of Korea, Bucheon, 420-743 Republic of Korea

**Keywords:** Acidic, EMP48-D, *Micrococcus luteus*, Novel enzyme

## Abstract

**Background:**

Lipases have emerged as essential biocatalysts, having the ability to contribute to a wide range of industrial applications. Microbial lipases have garnered significant industrial attention due to their stability, selectivity, and broad substrate specificity. In the previous study, a unique lipolytic bacterium (*Micrococcus luteus* EMP48-D) was isolated from tempeh. It turns out the bacteria produce an acidic lipase, which is important in biodiesel production. Our main objectives were to clone the acidic lipase and investigate its potential in biodiesel production.

**Result:**

In this study, the gene encoding a lipase from *M. luteus* EMP48-D was cloned and expressed heterologously in *Escherichia coli*. To our knowledge, this is the first attempt at the cloning and expression of the lipase gene from *Micrococcus luteus*. The amino acid sequence was deduced from the nucleotide sequence (1356 bp) corresponded to a protein of 451 amino acid residues with a molecular weight of about 40 kDa. The presence of a signal peptide suggested that the protein was extracellular. A sequence analysis revealed that the protein had a lipase-specific Gly-X-Ser-X-Gly motif. The enzyme was identified as an acidic lipase with a pH preference of 5.0. Fatty acid preferences for enzyme activities were C8 and C12 (p-nitrophenyl esters), with optimum temperatures at 30–40 °C and still remaining active at 80°C. The enzyme was also shown to convert up to 70% of the substrate into fatty acid methyl ester.

**Conclusion:**

The enzyme was a novel acidic lipase that demonstrated both hydrolytic and transesterification reactions. It appeared particularly promising for the synthesis of biodiesel as this enzyme’s catalytic reaction was optimum at low temperatures and was still active at high temperatures.

## Introduction

The discovery of enzymes has become a very important breakthrough in the biotechnology industry. The largest share of the enzyme market for industrial use is occupied by hydrolytic enzymes such as protease, amylase, amidase, esterase, and lipase [1]. In the past few decades, lipase (triacylglycerol acyl-hydrolase) has emerged as an important enzyme in biotechnology and is developing very rapidly [2]. This is due to the multifaceted nature of lipase, which allows its use in a variety of applications [3].

Lipase is also an enzyme that has abundant availability in nature and can be isolated from various microorganisms, animals, or plants [4]. Tempeh (originally written as tempe) is a traditional Indonesian fermented food produced by the fermentation of soybeans using *Rhizopus oligosporus.* Indonesian tempeh is an ecosystem with a high diversity of microorganisms [5], and some of these bacteria play a role in the hydrolysis of fats [6, 7]. During the process of fermentation of soybeans, hydrolysis of fat by microorganisms leads to an increased amount of free fatty acids in tempeh by 25–26% [8]. It suggests some other bacteria in association with tempeh are also capable of producing lipase.

Lipase (EC 3.1.1.3) in the enzyme classification system belongs to members of the carboxylic hydrolases [9]. Lipase catalyzes both the hydrolysis and synthesis of ester groups [10]. Lipase is one of the most important biocatalysts in a variety of industrial applications, i.e., food, detergent, pharmaceuticals, cosmetics, bioremediation, and oleochemicals. It covers around 5 to 10% of the enzyme market share in the world [2]. Some characteristics expected in industrial applications of lipases include stability at high temperatures, resistance to organic solvents, and the ability to be active in low pH conditions.

Acidic lipase (active at low pH) is very important in industrial applications, especially in biodiesel production [11, 12]. The composition of the substrate commonly used in biodiesel production is dominated by free fatty acids. The accumulation of free fatty acids causes the initial pH of the reaction to be very low, so alkaline biocatalysts will be more difficult and uneconomical to apply under these conditions [13]. Acidic lipase is the least studied type of lipase; so far, only a few lipases have been reported to show this characteristic. In this study, we succeeded in cloning acidic lipase-encoding genes from *Micrococcus luteus* EMP48-D. The gene has also been successfully expressed in *E. coli* BL21, and several analyses have been carried out about the characterization of the resulting lipases.

## Materials and methods

### Media, strains, and plasmids

Tributyrin and tricaprylin agar were used for detecting lipolytic activity. The media contain 1% (v/v) glycerin tributyrate or tricaprylate glycerin, 1 × gum arabic solution, 1% (w/v) tryptone, 0.5% (w/v) yeast extract, 0.5% (w/v) NaCl, and 2% (w/v) agar. Gum arabic solution (10 ×) contains 10% gum arabic, 200 mM NaCl, and 50 mM CaCl_2_. Nutrient (NB) agar or Luria Bertani (LB) agar were used for transformant verification and recoveries. For detecting lipolytic activity from the recombinant strain, trybutyrin and trycaprylin agar were treated with ampicillin (100 ppm), 25 mg/l X-galactosidase, and 50 mg/l isopropyl thiogalactopyranoside (IPTG). *E. coli* DH5ɑ was used as a cloning host for the gene encoding lipase. Cloning vectors were pGEM-T Easy plasmids, which contain the structural gene lipase EMP48-D. *E. coli* BL21 was used as an expression host. The pET15-b, pG-KJE8, pGro7, pKJE7, pG-Tf2, pTf16, and pUC19 were used as expression vector.

### Gene cloning and sequence analysis

**Fig. 1:**
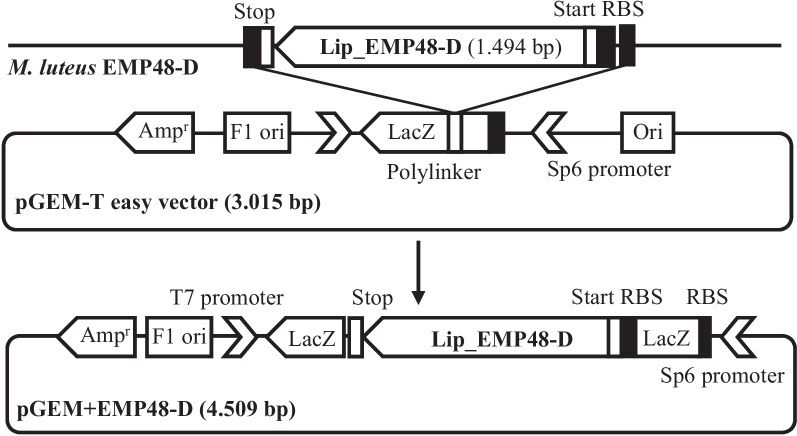
Schematic diagram of the cloning construct of Lip_EMP48-D into the pGEM-T Easy vector. Ori, origin of replication; RBS, ribosomal binding sites; Amp^r^, marker of ampicillin resistance

*M. luteus* EMP48-D isolates were isolated from tempeh [14]. DNA from *M. luteus* EMP48-D was extracted using the Wizard® Genomic DNA Purification Kit (Promega) with slight modifications. Lysozyme solutions (100 µg/ml) were added to optimize *Micrococcus* cell wall lysis. The gene-encoding lipase was amplified from genomic DNA with Lip013 primers: 5′-CCCCGACGCTAGCCGAG-3′ and 5′-CATCTGCATCCGAGAGACCG-3′. Amplicons were introduced into plasmid pGEM-T Easy using the TA cloning technique [15] and then transformed into *E. coli* DH5α (Fig. 1).

The size of the recombinant DNA carrying the lipase gene was confirmed using gel electrophoresis, while the sequencing of nucleotides was conducted using the AB® Genetic Analyzer 3130 (Applied Biosystem, USA). Lipase genes were amplified from recombinant plasmids using M13 primer (5′-GTAAAACGACGGCCAG-3′ and CAGGAAACAGCTATGAC) and Lip013 primer. Nucleotide sequence analysis was assisted using Geneious R.11.1.2 (Biomatters Ltd., NZ) and MEGA 6.06 (Pennsylvania State University, USA), and identification of homology was compared with sequences deposited in the database of NCBI GenBank (http://www.ncbi.nlm.nih.gov).

### Construction of expression vector and host-vector optimization

**Fig. 2:**
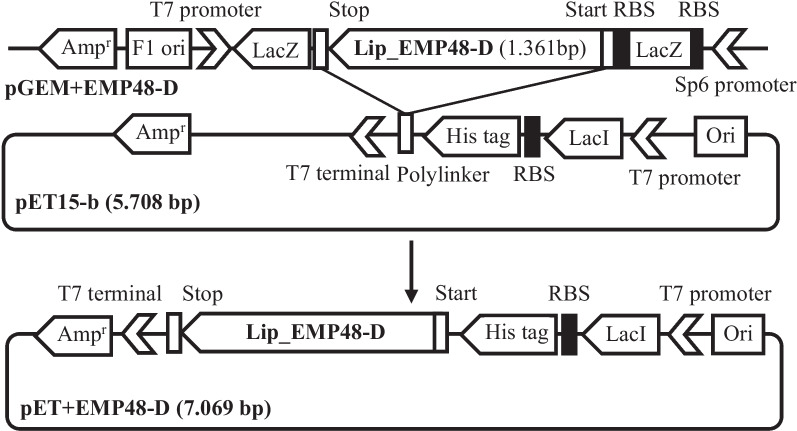
Schematic diagram of the expression construct of Lip_EMP48-D into pET15-b (expression vector). Ori, origin of replication; RBS, ribosomal binding sites; Ampr, marker of ampicillin resistance

The open reading frame (ORF) of the lipase gene was amplified with primers LiPML-*Nde*I 5′-GGAATTCCATATGGCCCCCGCACGCC-3′ and LiPML-*Xho*I 5′-CCGCTCGAGTCAGAACCACCCGCACGAGTC-3′. The predicted primers contain the nucleotides corresponding to the *Nde*I and *Xho*I sites used to target the gene in the right direction. The lipase gene (EMP48-D lipase) was ligated into an expression vector employing a double digest mechanism (Fig. 2). Recombinant plasmids were transformed into hosts described before using the heat shock method [16]. Transformants containing the recombinant plasmid were grown in LB medium containing chloramphenicol (35 μg/ml) and ampicillin (100 μg/ml) at 37°C to an OD600 of 0.6. The culture was then adjusted to 1 mM IPTG and incubation continued at 30°C for 24 h. Transformant cells were harvested by centrifugation at 10,000 × g for 30 min. The cells collected by centrifugation were washed two times and resuspended in the 10 mM Tris-HCl buffer (pH 8.0). Suspensions were sonicated to release intracellular protein, then the cell-free extract was centrifuged at 10,000 × g for 10 min to eliminate cell debris and assayed for lipase activity. The supernatant obtained from the first centrifugation was assayed for intracellular lipase activity.

### Molecular modeling of EMP48-D lipase

The initial alignment was carried out according to an automated web-based service from the ExPASy Proteomics server using FASTA, CLUSTALW, and T-Coffee. Protein structure prediction was generated semi-automatically using a homologous structure modeling program on SWISS-MODEL servers (https://swissmodel.expasy.org) [17]. The model was then visualized via the visual molecular dynamic (VMD) software v.1.9.3 (University of Illinois, USA). The template used for the modeling was the crystal structure of lipase A from *C. antarctica* CalA (2veo.1.A). The active enzyme site was predicted using Geneious R.11.1.2 software (Biomatters Ltd., New Zealand). Lipase functional components were predicted using phobius web-based prediction servers (http://phobius.sbc.su.se/cgi-bin/predict.pl) and SignalP 4.1 (http://www.cbs.dtu.dk/services/SignalP).

### Determination of lipase activity

Lipase activity was measured spectrophotometrically using p-nitrophenyl (p-NP) ester as a substrate [18]. The reaction mixture for the standard assay (1.0 ml) was prepared with 2-mM p-NP-laurate (C12) in acetonitrile, 150-mM citrate-phosphate buffer (pH 5.0), and 10-μL crude enzyme. The blank reaction was carried out identically to the assay mixture, excluding the enzymes. The mixture was incubated at 37°C for 5 min, and the amount of p-nitrophenol released from p-nitrophenyl ester was measured at 405 nm. Protein quantities were estimated with bicinchoninic acid (BCA) kits (Thermoscientific) and bovine serum albumin (BSA) as a protein standard according to manufacture procedures. One unit of lipase activity (U) was defined as the amount of a particular enzyme that catalyzes the conversion of 1 μmol of p-NP per minute, while specific enzyme activity is expressed as a unit of enzyme per mg of protein (U/mg).

### SDS-PAGE and zymogram

Sodium dodecyl sulfate-polyacrylamide gel electrophoresis (SDS-PAGE) was carried out based on the method proposed by Laemmli [19] with slight modifications. Zymography was carried out following the SDS-PAGE work procedure. The staining step was replaced by soaking the SDS-PAGE gel in 1% Triton X-100 for 2 × 30 min, then rinsed with distilled water for 60 min. The gel was then placed on a tributyrin agar medium and incubated at 37°C for 8 h, or until the clear zone was visible. The clear band on tributyrin media was compared to molecular weight markers on the SDS-PAGE gel to obtain the range of molecular weight (MW).

### Lipase characterization

Substrate specificity for nitrophenyl ester was analyzed under standard conditions using p-nitrophenyl butyrate (C4:0), p-nitrophenyl caproate (C6:0), p-nitrophenyl caprylate (C8:0), p-nitrophenyl decanoate (C10:0), p-nitrophenyl laurate (C12:0), p-nitrophenyl palmitate (C16:0), and p-nitrophenyl stearate (C18:0). The optimal temperature for enzyme activity was tested at various temperatures within the range of 20 to 100°C. Thermostability was determined by measuring the residual activity after incubating the enzyme at temperatures of 50°C, 65°C, and 80°C for 240 min. The optimum pH was determined at various pH within the range of 3.0 to 10.0 at 37°C. The pH stability was tested by measuring the residual activity after incubating the enzyme at pH of 4.0, 7.0, and 9.0 for 180 min. Stability in organic solvents was determined by measuring the residual activity after incubation of the enzyme with 50:50 (v/v) organic solvents, i.e., methanol, ethanol, butanol, 2-propanol, acetonitrile, n-hexane, and n-heptane, at 37°C for 180 min. The effect of metal ions was measured under standard conditions by the addition of various metal salt solutions, i.e., FeSO_4_, ZnCl_2_, MgCl_2_, CaCl_2_ (0.001 M); KCl, MnCl_2_ (0.002 M); and NaCl (0.01 M). The effects of various detergents or inhibitor agents were investigated by the addition of 0.5% sodium dodecyl sulfate (SDS), 1% Tween 80, 1% Tween 20, 0.5% EDTA, 1% Triton X-100, and 2% glycerol. The activity measurements were made through three independent repetitions for each variable investigated under the standard assay conditions described before.

### Transesterification reaction

Transesterification reactions were carried out in 10 ml vials in an incubator at constant temperature with continual shaking. The reaction was carried out as follows: 2 mL of enzyme (50 U) was added to the free fatty acid solution. A free fatty acid solution was created by dissolving palmitate acid or lauric acid in methanol, which acted as reactant and solvent, respectively. The molar ratio of free fatty acid to methanol was 1:3 [20]. Incubation conditions were maintained at 50°C with agitation at 225 rpm for 5 h. The reaction product was separated into two phases: the top phase contained excess methanol and water formed during the reaction, while the crude fatty acid methyl ester phase was at the bottom. The fatty acid methyl ester (FAME) phase was taken off at the bottom and passed into a centrifuge tube for further centrifugation to remove traces of methanol. The residual free fatty acids on the FAME were required for further analysis. The amount of free fatty acids in the crude fatty acid methyl ester was determined by titration with NaOH 0.05 mol/L for maximum conversion. The percent conversion of free fatty acids was defined as the amount of free fatty acids successfully converted to fatty acid methyl ester.

## Result

### Gene cloning and sequence analysis

Ligation between the EMP48-D lipase gene and the pGEM-T vector produced 4.4 kbp of recombinant DNA. PCR amplification using Lip013 primers produced 1.4 kbp DNA fragment, while amplification using M13 primers produced 1.7 kbp. The attachment of the M13 primer was located at 176 and 66 bases upstream and downstream of the polylinker (MCS), respectively, so amplification using this primer will produce fragment sizes about 242 bp longer than the insert. Restriction of the recombinant plasmid using *Eco*RI produced 1.4 and 3.0 kbp DNA fragments, similar to the size of the DNA insert and the pGEM-T easy vector.

PCR amplification of the *M. luteus* EMP48-D chromosome using Lip013 primers produced 1.414 bp DNA fragment, covering the whole open reading frame (ORF) with an additional 37 bases before the start codon (−37) and 21 bases after the stop codon (1.393). The ribosome binding site (RBS) is located at −15 to −11 (5 bp), while the transcription initiation site (promoter) is located at −35 to −21 (15 bp). The ORF size was 1356 bp, which encodes 451 amino acid residues (AA). The ORF sequences carry 93 nucleotides (31 AA) of peptide signal that indicates the protein might be transferred outside the cell [21]. The amino acid sequence showed similarities to lipases from *Micrococcus luteus* trpE16, *Streptococcus pneumonia*, and *Micrococcus lylae* (Table 1).

**Table 1:** Results of the blastP EMP48-D amino acid lipase sequence (451 AA)

No.	Description	*E* value	Ident.	Accession
1.	Lipase [*Micrococcus luteus* strain trpE16]	0.0	96 %	WP_073116234.1
2.	*Secretory* lipase [*Streptococcus pneumonia*]	0.0	96 %	CVM40669.1
3.	Lipase [*Micrococcus lylae*]	2e−157	69 %	WP_102214108.1
4.	Lipase [*Micrococcus terreus*]	2e−103	57 %	WP_091698861.1
5.	Lipase [*Arthrobacter* sp. strain SW1]	2e−96	53 %	WP_070348929.1
6.	Lipase [*Streptomyces* sp. strain CNQ329]	7e−92	53 %	WP_027772499.1

### Expression and host-vector optimization

PCR amplification using LiP-ML primers that were added with the *Nde*I and *Xho*I recognition sites produced a DNA fragment of 1375 bp. DNA fragments were partially cleaved with *Nde*I and *Xho*I, and the resulting DNA fragments (1.361 bp) were inserted into vectors and transformed into *E. coli*. Transformants were selected on tributyrin agar plates; DNA insertion was indicated by white colonies and clear zone formation. However, both *E. coli* hosts (white colonies) containing either vector showed no lipolytic activity (clear zone) in media selection. For confirmation purposes, the DNA insert was sequenced, and the Lip-EMP48-D-containing DNA sequences were found in the correct size and direction.

The wild-type strain of EMP48-D was an extracellular lipase harboring the highest activity of 17.02 ± 3.01 U/mg (Table 2). As mentioned above, the presence of the plasmids carrying Lip-EMP48-D in *E. coli* did not lead to any lipolytic activities on selection media. So, we tried to extract the cells, where the supernatant contained the soluble proteins and the pellet contained the insoluble cell fractions. The supernatant still did not show any lipolytic activity on tributyrin agar, whereas the pellet from a specific combination (host-vector) showed lipolytic activity. Pellets from recombinant *E. coli* DH5α with pGEM-T easy vector and pUC-19 did not show lipolytic activity on tributyrin agar. Fortunately, pellets from recombinant *E. coli* BL21 with pET15-b vectors and origami variants successfully exhibited a clear zone on tributyrin agar. Surprisingly, the *E. coli* BL21 with pET15-b showed a much larger clear zone on tricaprylin plates as well as lipolytic activities than did the *E. coli* BL21 with origami vectors (Table 2).

**Table 2:** Optimization of host and vector systems in the expression of lipase EMP48-D

Host	Vector	Clear zone index^a^	Pellet activity (U/mg)
Wild-type	n/a	1.25	17.02 ± 3.01
*E. coli* DH5α	pGEM-T Easy	n/a	2.11 ± 1.09
	pUC-19	n/a	0.93 ± 0.07
***E. coli* BL21 (DE3)**	**pLysS + pET15-b**	**1.0**	**15.02 ± 2.33**
	pET15-b	0.95	14.47 ± 1.99
*E. coli* BL21 with Origami variants	pG-KJE8 + pET15-b	0.75	10.33 ± 2.88
	pGro7 + pET15-b	0.45	7.65 ± 1.56
	pKJE7 + pET15-b	0.7	10.02 ± 0.98
	pG-Tf2 + pET15-b	0.65	8.57 ± 2.55
	pTf16 + pET15-b	0.4	5.35 ± 1.62

^a^The clear zone index was measured after 24 h of incubation on tributyrin agar and was calculated as the ratio of the diameter of the clear zones to the diameter of colonies

The activity was expressed as the mean ± standard deviation

### SDS-PAGE and zymogram

**Fig. 3:**
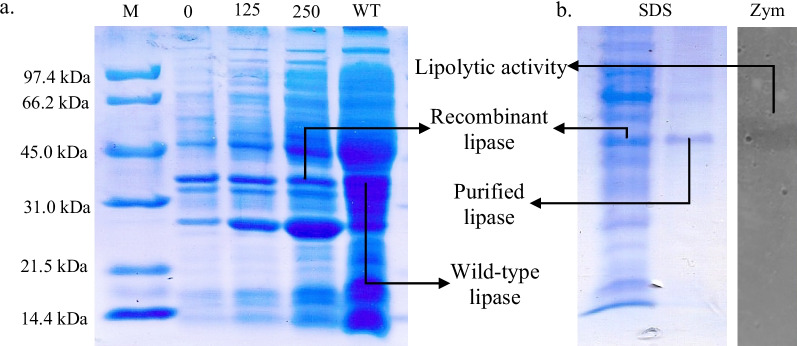
**a** SDS-PAGE and **b** zymogram of insoluble protein (pellet) of EMP48-D recombinant cells. (M) protein molecular weight markers; (0) crude extract from non-induced recombinant *E. coli*; (125) pellet from 125 µM IPTG-induced recombinant *E. coli*; (250) pellet from 250 µM IPTG-induced recombinant *E. coli*; WT, pellet from olive oil-induced wild-type strain; (SDS) partially purified EMP48-D lipase on SDS-PAGE; (Zym) lipolytic activity of the lipase on tributyrin substrates

The samples were subjected to SDS-PAGE. While the soluble fraction (supernatant) revealed no protein bands compared to the wild-type control, the pellet fraction revealed one IPTG induced protein bands (Fig. 3a). The SDS-PAGE showed the formation of the induced bands about 40 kDa. This size was similar to the predicted size about 40 kDa (1356 bp). Then, the protein band was confirmed using zymogram (Fig. 3b). The zymograph showed a clear zone formation at a band of approximately 40 KDa. This preference just confirmed the actual size of the protein was definitely about 40 KDa.

### Open reading frame of lipase sequence analysis

**Fig. 4:**
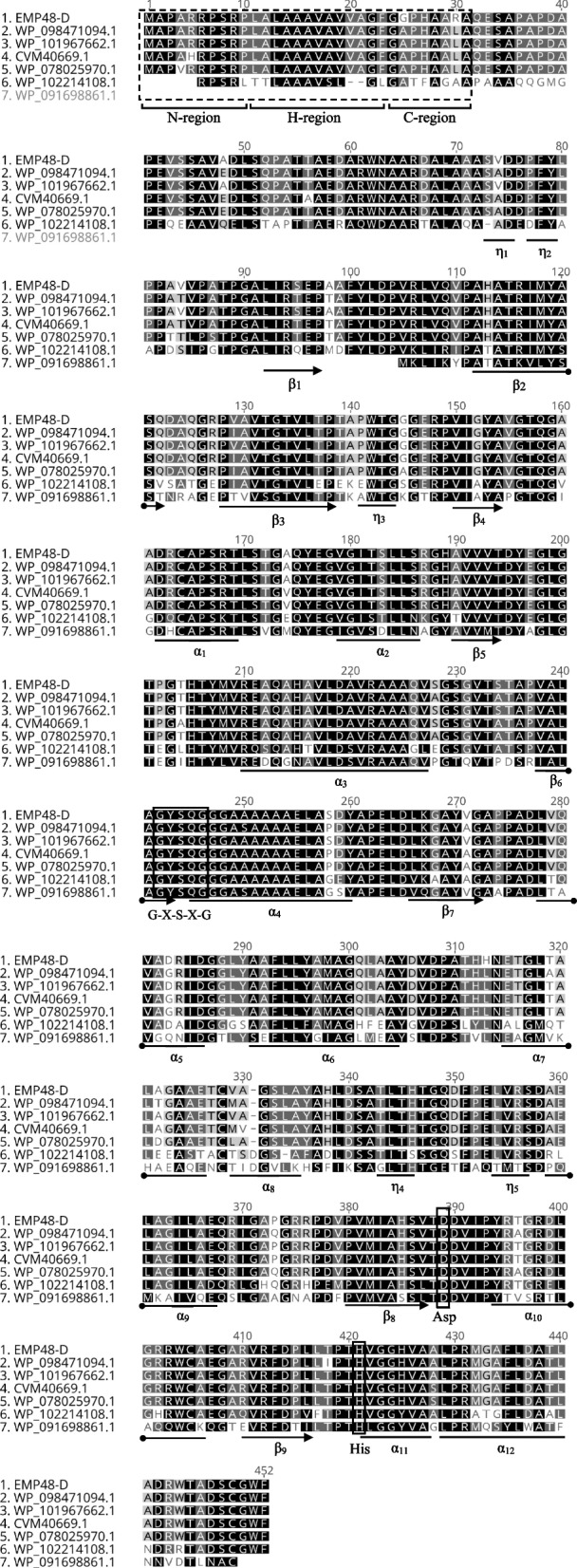
Multiple alignments of amino acid sequences between EMP48-D lipase and other closely known lipases have been reported for the *Micrococcaceae bacterium* JKS001869 (WP_098471094.1), *M. luteus* (WP_101967662.1), *S. pneumoniae* (CVM40669.1), *Micrococcus* sp. KBS0714 (WP_078025970.1), *Micrococcus lylae* (WP_102214108.1), and *Micrococcus terreus* (WP_091698861.1). The alpha helix, beta sheet, and 310-helices are identical to *α*, *β*, and *η*, respectively

The whole ORF of the lipase gene carried a nucleotide sequence of 1.356 bp, which encodes 451 amino acid residues (Fig. 4). The nucleotide sequence was dominated by high-GC nucleotides with 78% GC content. Amino acid sequences were analyzed using SignalP 4.1, which showed the presence of signal peptides (31 AA), and a cleavage site was detected between Ala31 and Gln32. It was a typical peptide signal found in most gram-positive bacteria [22]. Analysis using Phobius web-based prediction server revealed the peptide signal sequence consists of 10 residues of the N-Region (1–10), 13 residues of the H-Region (11–13), and 8 residues of the C-region (24–31) (Fig. 4). The peptide sequence from 32 to 451 prepares a mature lipase with the characteristics of a non-cytoplasmic transmembrane topology [23]. The presence of a signal peptide and transmembrane topology in amino acid sequences shows the potential for EMP48-D to be expressed outside the cell. The conserved amino acid domain of the Gly-X-Ser-X-Gly motif was found in EMP48-D lipases (X can be replaced with any amino acid). This motif is found in almost all amino acid sequences that make up lipase and esterase enzymes; some carry the G-D-S-L motif [24]. Serin 244 (ser244), aspartate 388 (Asp388) and histidine 350 (His350) are positioned in a catalytic triad-like configuration. The serine is part of a Gly-X-Ser-X-Gly consensus sequence (where X represents tyrosine and glutamine, respectively). Ser244 is located at the apex of the nucleophilic elbow between strand β6 and helix α4 (Fig. 4). The role of the side chain of Ser244 would be to anchor the protein backbone in a position favorable for an oxyanion hole [25].

### Molecular modeling of 3D lipase structure EMP48-D

**Fig. 5:**
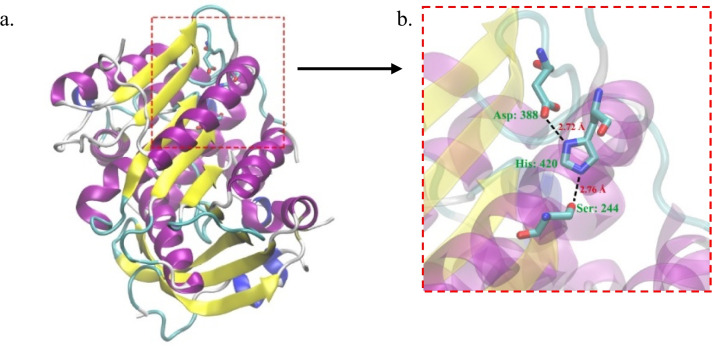
3D model of EMP48-D lipase. **a** The α-helix, β-sheet, random coil, and beta turn are shown in cartoon form in purple, yellow, and cyan, respectively. **b** The catalytic triad (Ser 244, Asp388 and His420) are zoomed in

Lipase EMP48-D has a globular shape with dimensions of 35 Å × 36 Å × 42 Å. The core structure consists of nine stranded β-sheet which are surrounded by twelve α-helices (similar to ORF analysis in Fig. 4). There are five α-helices on one side of the β-sheets and seven α-helices on the other side (including those consisting of only four amino acid residues). The secondary structure of lipase EMP48-D has an α/β hydrolase fold similar to that present in all lipases (Fig. 5). As no crystal structure of a *Micrococcus* lipase is available, we have submitted a primary sequence of EMP48-D lipase to the Swiss-Model server for automatic modeling (http://www.expasy.org/spdvb). Modeling by comparison to the protein structures of the *C. antarctica* (2veo.1.A) and *C. antarctica* (3guu.1.A) lipases in PDB format was applied to generate a 3D model of the EMP48-D lipase. The resulting protein structures of the enzymes were then viewed and labeled using VMD software, as shown in Fig. 5.

### Classification of EMP48-D lipase

**Fig. 6:**
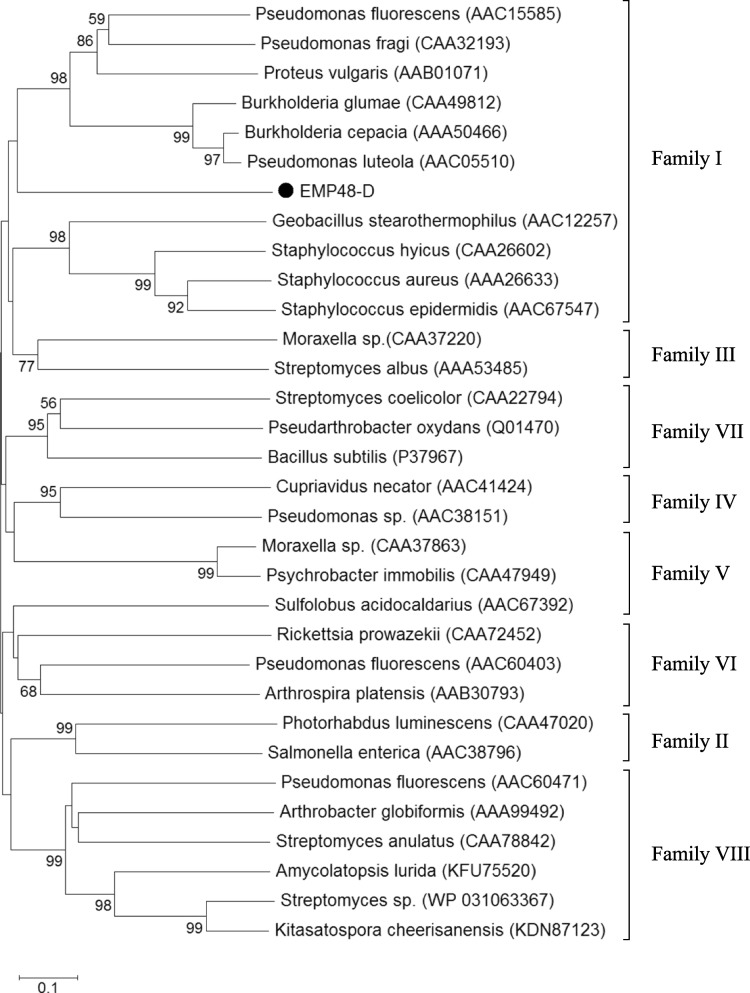
Phylogenetic analysis based on amino acid sequences of EMP48-D lipase and other related esterase/lipase families [26]. A phylogenetic tree was constructed by the neighbor-joining method (1000 × bootstrap) using MEGA 6.0 software

**Fig. 7:**
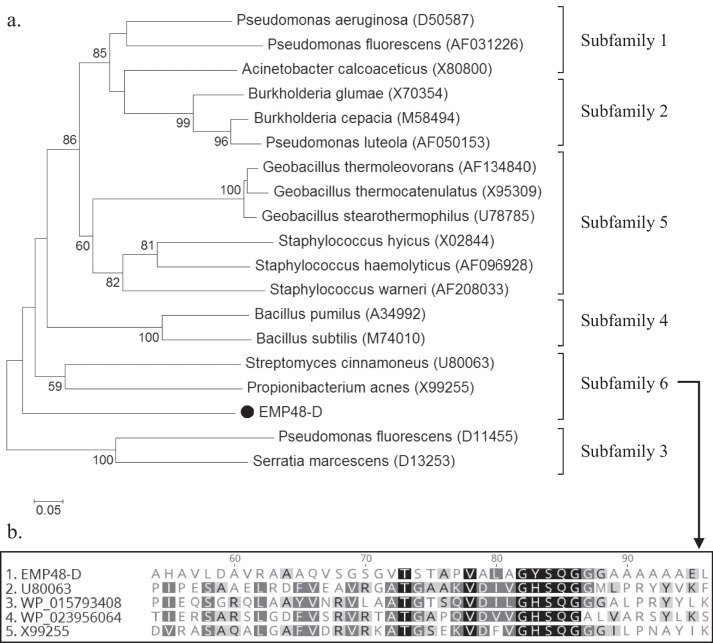
**a** Phylogenetic analysis of EMP48-D lipase and other related lipases from family I (subfamilies 1 to 6). The phylogenetic tree was constructed by the neighbor-joining method (1000 × bootstrap) using MEGA 6.0 software. **b** Multiple sequence alignment of EMP48-D lipase and known related lipases from subfamily I.6. U80063: lipase from *Streptomyces cinnamoneus*, WP_ 015793408: lipase from *Catenulispora acidiphila*, WP_023956064: lipase from *Williamsia* sp, and X99255: lipase from *Propionibacterium acnes*

To investigate how EMP48-D lipase was related to known esterases/lipases, the phylogenetic relationship was analyzed based on the esterase/lipase classification. Bacterial esterases/lipases have been classified into eight different families based on their amino acid sequences and biochemical properties [26]. A phylogenetic tree was constructed from the aligned sequences by the neighbor-joining method based on *p*-distances using CLUSTALW. The tree presented in Fig. 6 is a bootstrap consensus after 1000 repetitions. The phylogenetic tree showed that EMP48-D lipase was closely related to the proteins within family I of lipolytic enzymes. Lipolytic family I is the most represented family and is divided again into six subfamilies. On the basis of the amino acid sequence comparison and phylogenetic analysis, EMP48-D belonged to subfamily I.6 (Fig. 7). Lipase from *M. luteus* EMP48-D revealed a closer relationship with lipase from *Streptomyces cinnamoneus* and *Propionibacterium acnes*. Lipase from *Catenulispora acidiphila* (WP_015793408) and *Williamsia* sp. (WP_023956064) are some other members of subfamily I.6 [27]. Lipases from families I generally share a Gly-X-Ser-X-Gly consensus sequence [28]. Lipases categorized as subfamily I.6 not only demonstrate the usual pentapeptide Gly-X-Ser-X-Gly but also, more specifically, share a Gly-X-Ser-Gln-Gly consensus sequence (Fig. 7b). It was also interesting to know that all known members of subfamily I.6 belong to the phylum *Actinobacteria*.

Multiple sequence alignment analysis of lipase from *M. Luteus* EMP48-D also showed a closer relationship to the lipase from subfamily I.3 (Fig. 7a), but the biochemical properties of lipase from subfamily I.3 commonly possessed a higher molecular mass (*P. fluorescens*, 50 kDa; *S. marcescens*, 65 kDa) and the absence of an N-terminal signal peptide. Lipase from *M. luteus* EMP48-D has a molecular mass of about 40 kDa. It was close to the molecular mass of lipase from *P. acnes* about 33 kDa and *S. cinnamoneus* about 26 kDa [29, 30]. Lipases from subfamily I.3 usually lack the N-terminal signal peptide and amino acid cysteine (Cys) which existed in EMP48-D lipase. Angkawidjaja and Kanaya [28] stated that lipases from subfamily I.3 usually belong to members of gram-negative bacteria.

### Specific substrate and stability to organic solvents

**Fig. 8:**
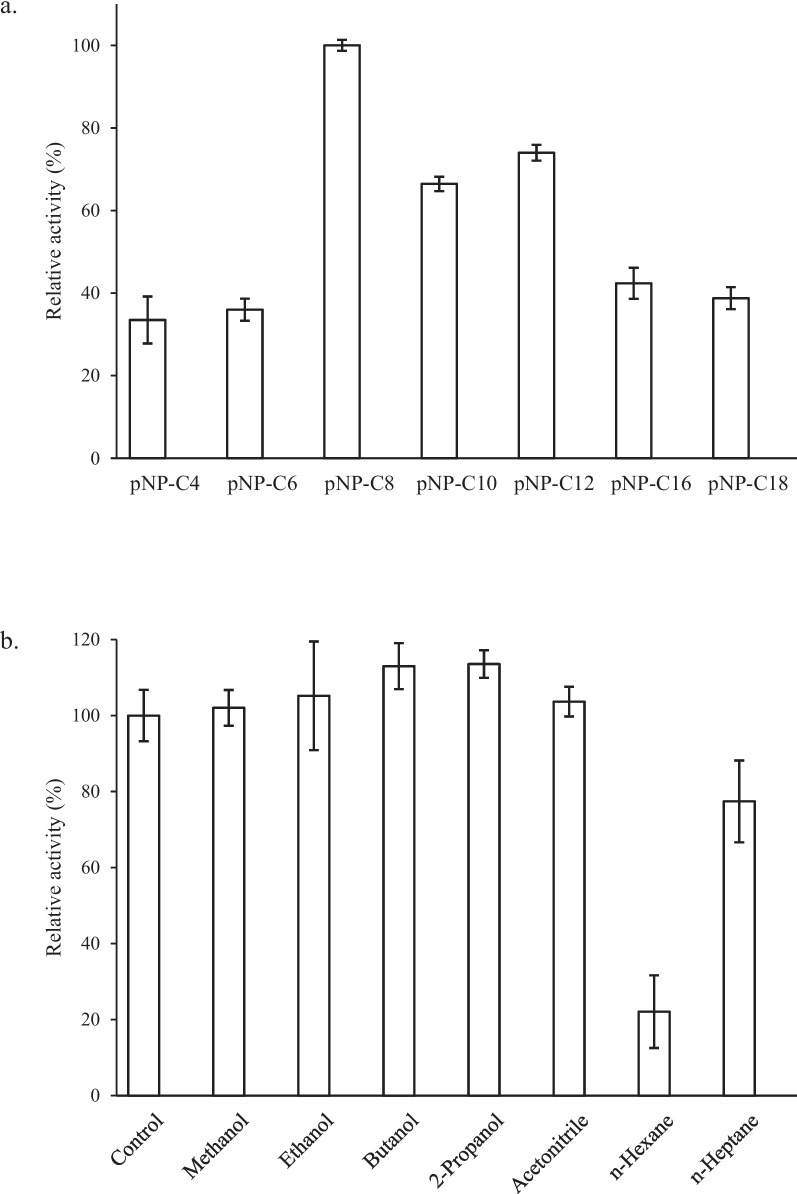
**a** Substrate specificity of EMP48-D lipase against various p-nitrophenyl esters. **b** lipase stability in various organic solvents. All reactions were performed in triplicate

The activities of the EMP48-D lipase towards various p-nitrophenyl esters were investigated (Fig. 8a). EMP48-D lipase showed the highest relative activity towards p-nitrophenyl caprylate (100%) among the substrates investigated. The enzyme also showed good activity towards p-nitrophenyl laurate (74%) and p-nitrophenyl caproate (66.4%). Enzyme specificity toward p-nitrophenyl caproate (66.4%). p-nitrophenyl palmitic (42.4%), p-nitrophenyl stearate (38.8%), and p-nitrophenyl butyrate (33.5%) showed the lowest activities. Short-chain p-nitrophenyl esters were hydrolyzed poorly, while enzyme specificity towards long-chain p-nitrophenyl esters strongly suggests that EMP48-D lipase is a true lipase [31].

The stability of the crude extracts toward p-nitrophenyl laurate substrates and various organic solvents was examined. The effect of various organic solvents on the stability of EMP48-D lipase is shown in Fig. 8b. The highest activity was retained in the presence of butanol (113%), 2-propanol (113.5%), ethanol (105.2%), methanol (102%), and acetonitrile (103.7%) compared to the control. In contrast, n-heptane and n-hexane decreased the lipase activity to 77.4% and 22.1%, respectively.

### Effects of metal ions, detergents, and inhibitors on lipase activity of EMP48-D

**Fig. 9:**
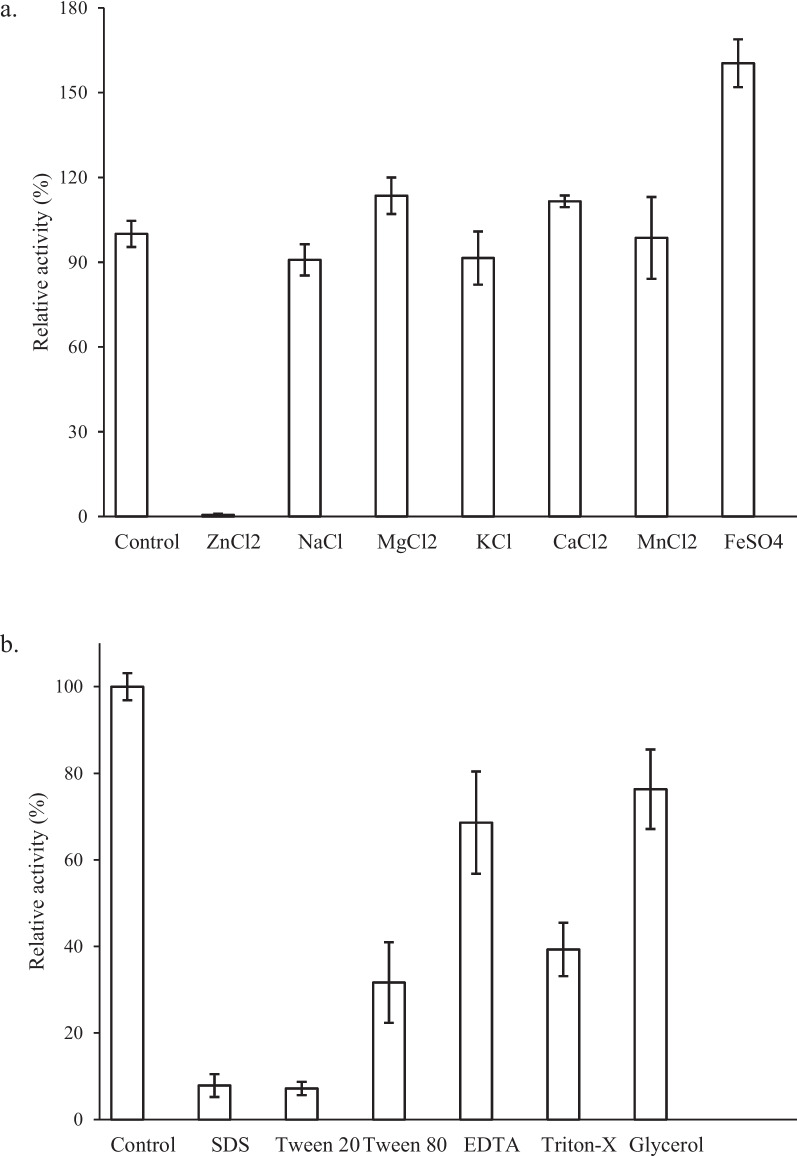
**a** Effect of various metal ions on EMP48-D lipase activity. **b** Effect of various detergents and certain inhibitor agents on EMP48-D lipase activity. All reactions were performed in triplicate

The effect of various metal ions on the activity of EMP48-D lipase is shown in Fig. 9a. EMP48-D lipase activity was significantly stimulated in the presence of Mg^2+^ (113.5%) and Ca^2+^ (111.5%). Other metal ions tested, i.e., Na^+^ and K^+^, had little effect on the lipase activity at 90.8% and 91.5%, respectively. The presence of Zn^+^ ions almost nullified the activity of the lipase (0.5%). Conversely, the Fe2^+^ ion greatly increased the activity of lipase (160.4%). The fact that the activity was not significantly inhibited by EDTA (66.6%) suggests that the lipase is not a metalloenzyme. The effects of various surfactants, oxidizing agents, and detergent ingredients on lipase activity are depicted in Fig. 9b. A high percentage of the lipase activity was lost in the presence of most detergents. This percentage was down to 39.3%, 31.6%, and 7.2% in the presence of Triton X-100, Tween 80, and Tween 20, respectively. A high loss of lipase activity was also found in the presence of SDS (7.9%). Glycerol showed a deactivating effect but was not considerably significant.

### Temperature and pH optimum and EMP48-D lipase stability

**Fig. 10:**
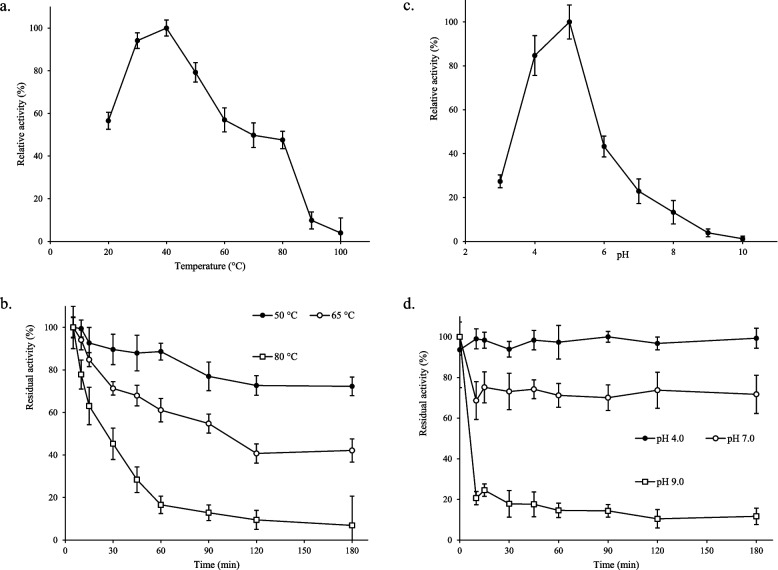
Effects of temperature and pH on the activity and stability of EMP48-D lipase. **a** The enzyme activity was measured at various temperatures at pH 5.0. **b** The enzyme stability was measured at various temperatures at pH 5.0 for 180 min. **c** The enzyme activity was determined at various pHs at 37°C. **d** The enzyme stability was measured at various pHs at 37°C for 180 min

The temperature optimum for the enzyme was observed at 40°C with pNP-laurate as substrate (Fig. 10a). The maximum activity was 14.79 U/mg, and it retained 45.25 % of its activity at 80°C. This suggests that the lipase produced by *M. luteus* EMP48-D is thermotolerant. The enzyme was stable from 50 to 65°C for 100 min; however, enzyme activity was drastically reduced at 80°C. The enzyme was almost completely stable at 50°C and 65°C for 60 min, while the activity was reduced to 79.1% and 37.5% after 180 min of incubation, respectively (Fig. 10b).

The effect of pH on the lipase activity of *M. luteus* EMP48-D was investigated for pH values from pH 3.0 to 10.0, as shown in Fig. 10c. The enzyme displayed maximum activity at pH 5.0 and retained 84.7% of its activity at pH 4.0. The maximum activity was 15.21 U/mg and was completely stable when incubated at room temperature for 180 min. Lipase from *M. luteus* EMP48-D was able to tolerate a low pH range, suggesting this lipase was an acidic lipase. However, beyond pH 5.0, the activity rapidly dropped, reaching a value of 1.2% at pH 10.0. The stability of the enzyme remained relatively stable within the pH range, ranging from 4.0 to 5.0, and dropped to lower values of stability of 71.7% and 11.6% at pH 7.0 and 9.0 after 180 min of incubation, respectively (Fig. 10d).

### Transesterification reaction

Lipase EMP48-D and lipase T.1.2 (lipase T.1.2 was provided by PT. Wilmar Benih Indonesia) were compared regarding the conversion of fatty acids from caprylic acid (C:8) and palmitic acid (C:16), as shown in Table 3. A comparison between EMP48-D and T.1.2 was also evaluated using the same activity units (20 U). The use of the same activity unit values resulted in employing different amounts of EMP48-D and T.1.2 in esterification reactions of free fatty acids. Reactions were carried out at 50°C, considering the melting point of palmitic acid. The methanol was added in a stepwise manner to avoid high amounts of methanol in the reaction medium at the beginning of the reaction. EMP48-D presented a higher conversion than T.1.2 in the substrates of caprylic acid and palmitic acid. EMP48-D in caprylic acid and palmitic acid obtained a conversion of 64.7% and 77.2%, respectively. Lipase T.1.2 was less active for the esterification reaction than lipase EMP48-D. The free fatty acid conversion values were 55.6% and 61.3% in the reactions with caprylic acid and palmitic acid, respectively. Thus, no significant increase in conversion was observed when the reaction time increased from 5 to 24 h, which is interesting from an economic point of view.

**Table 3:** Lipase-catalyzed transesterification of EMP48-D and T.1.2 lipases using caprylic acid and palmitic acid as the substrates

Enzyme	Substrate	Incubation time (hour)	Final FFA (%)	FA conversion (%)
EMP48-D	Caprylic acid	5	22.88 ± 0.70	61.98 ± 1.16
		24	21.25 ± 0.61	64. 69 ± 1.01
	Palmitic acid	5	18.15 ± 1.75	70.59 ± 2.83
		24	14.04 ± 0.32	77.25 ± 0.51
Lipase T.1.2	Caprylic acid	5	26.98 ± 1.31	55.18 ± 2.18
		24	26.71 ± 1.22	55.62 ± 2.03
	Palmitic acid	5	24.553 ± 0.50	60.223 ± 0.81
		24	23.868 ± 0.22	61.333 ± 0.35
Control	Caprylic acid	-	60.194 ± 0.72	-
	Palmitic acid	-	61.727 ± 0.88	-

The activity was expressed as the mean ± standard deviation

## Discussion

Lipases from microorganisms have been discovered since the 1950s, and studies on their application in the bio-industry have continuously progressed. Many lipases from microbial sources are already being designed or purposely engineered in various commercial processes. However, there are still some cultivable microorganisms that are potential sources of novel lipases that have not yet been explored. In this respect, lipase properties from *Micrococcus luteus* are the only ones that have never been reported. This is supposed to be the first report of the cloning and characterization of recombinant lipase from the *Micrococcus luteus* strain. Studies about *Micrococcus luteus* lipase have limited the detection of lipase activities in strains derived from an artisanal raw ewe’s milk cheese [32]. The closest report might be the study about lipase characterization from a wild-type strain of *Micrococcus freudenreichii*, which is optimal at pH 8.0–8.5 [33].

The bacterial strain *Micrococcus luteus* EMP48-D was isolated from tempeh, a traditional fermented food from Indonesia. Several studies have reported the presence of *Micrococcus* strains in tempeh [34]. Using the direct PCR cloning technique, we succeeded in cloning the lipase gene, but nothing showed a clear zone on media selection. This might be related to host incompatibility due to transcription or translation of the lipase in *E. coli* cells [35]. To overcome this problem, we figured it out with detection using the supernatant, which contained the soluble proteins, and the pellet, which contained the insoluble cell fractions. Lipase activity was found essentially in the pellet fraction, which suggested that the lipase is associated with the cell wall fraction or that inclusion bodies were formed [36]. SDS-PAGE revealed that the apparent molecular weight of this protein (40 kDa) is similar to the predicted molecular weight of 40 kDa. The molecular weight (MW) of a protein is usually predicted based on its amino acid composition (AA).

Multiple sequence alignment of sequences revealed that EMP48-D lipase contained a conserved Gly-X-Ser-X-Gly sequence that is characteristic of all lipases [37]. A phylogram constructed based on multiple sequence alignment analysis revealed that EMP48-D lipase was closely related to a lipase from *Micrococcus luteus* and *Streptococcus pneumonia*. The 3D structure of proteins can be predicted by using homology modeling, which uses experimentally determined protein structures as templates to predict the 3D structure of a target protein based on target-template alignment [38]. The EMP48-D-modeled lipase is a monomer folded into α/β domain. It consists of nine stranded β-sheets surrounded by twelve α-helices. The same structure is shared by other lipase enzymes of mammalian and bacterial origin, whereas the number of α-helices and β-sheet differs from one species to another [39]. Based on the classification proposed by Arpigny and Jaeger [26], the biophysical properties and multiple sequence alignment of EMP48-D lipase showed that it belonged to the subfamily I.6 of bacterial lipases. The molecular mass of EMP48-D was also in the range of members of subfamily I.6 around 30 kDa [26].

The lipase displayed higher activity on the substrate of long-chain fatty acid esters (C8:0–C12:0) but low activity towards short-chain fatty acid esters (C4:0–C6:0). This preference for these substrates indicated that EMP48-D was a true lipase [18]. The specific activity of lipase EMP48-D (15.02 ± 2.33 U/mg) is relatively poor compared to that of most bacterial enzymes that are commercially available. Special characteristics of microbial enzymes include their capability and appreciable activity under abnormal conditions, mainly temperature and pH. Lipase from *M. luteus* EMP48-D has unique properties, such as being active in the low pH range and having high-temperature stability. Optimal activity for EMP48-D lipase was found to occur in conditions similar to those in which the source organism was isolated (tempeh). The optimum pH for tempeh fermentation is in the range of pH 5.0 to pH 6.7 [40] and EMP48-D lipase showed the highest levels of activity around these conditions (pH 5.0). The high activity and pH stability in acidic conditions indicated that the EMP48-D protein was an acidic lipase, unlike many other bacterial lipases (alkaline). EMP48-D lipase was also found to have an optimum temperature of 40°C and be relatively stable in the range of 30 to 65°C. This is consistent with the temperature range of 30 to 49°C [40]. The stability of EMP48-D lipase in different organic solvents was variable. EMP48-D lipases were more stable in the presence of polar (protic) solvents than nonpolar solvents. Many studies stated that the catalytic activity and stability of enzymes are affected by the presence of organic solvents; often, such changes in enzyme properties were correlated to polarities in terms of structural integrity between the active site and the substrate [41]. The effects of denaturants and detergents on EMP48-D lipase activity were also variable, as SDS, Tween 20, and Tween 80 were observed to be inhibitory, while glycerol and Triton X-100 showed an insignificant effect on enzyme activity. Tween 20 and Tween 80 were reported to be similar to p-nitrophenyl laurate and p-nitrophenyl oleic, respectively, which are both substrates for lipase categories [42, 43]. Furthermore, they appear to competitively inhibit the catalytic domain of EMP48-D lipase, suggesting that Tweens could be a more desirable competitive inhibitor of EMP48-D lipase than other evaluated agents. Tween 20 is more eligible to be a substrate for competitive inhibition than Tween 80, which signifies that lipase prefers C12 (Tween 20) over C18 (Tween 80). SDS inhibited strongly the activity of EMP48-D lipase. SDS might act upon the disulfide linkage of the enzyme and so cause inactivation of the enzyme [44]. On the basis of their effect on EMP48-D activity, the metal ions could be categorized as Na^+^, K^+^, and Zn^+^, which were found to reduce the enzyme activity, and Ca^2+^, Mg^2+^, Mn^2+^, and Fe^2+^, which were found to enhance the enzyme activity. There is no consistent trend reported regarding the effect of metal ions on lipase activity [45]. The effect varies with different kinds of metal; even the same metal is found to affect the lipase activity in a different manner if the lipase source has been varied. However, enzyme activity is not significantly affected by metal chelating agents (EDTA), which indicates that EMP48-D lipase activity is independent of metal ions.

Lipase-catalyzed transesterification for biodiesel production has been an active area of research and shows great potential to generate an environmentally friendly and economic fuel in the future. To study prospective scale-up for biodiesel production by enzymatic reactions, and in view of the fact that commercial lipases are purchased at high prices, investigating alternative lipases is essential. EMP48-D lipase was found to be relatively more active for the transesterification reaction than the other lipase (T.1.2). In addition, EMP48-D lipase showed better transesterification performance in the presence of palmitic acid than caprylic acid. Palmitic acid is the most abundant resource, consisting of PFAD, a byproduct of the palm oil industry [46]. Thus, the use of EMP48-D lipase may offer some advantages in relation to chemical catalysts for biodiesel production.

## Conclusion

A promising novel lipase from *Micrococcus luteus* EMP48-D has been successfully cloned and characterized. The lipase structural model fits the α/β hydrolase fold for hydrolytic enzymes and is classified as subfamily I.6. The lipase demonstrates very high activity towards C8:0 fatty acids and can still perform at fairly high temperatures. EMP48-D lipase was discovered as an acidic lipase, and its transesterification ability gives it a promising prospect for meeting the needs of most industries, especially biodiesel production.

## Data Availability

Not applicable.

## Declarations

Ethics approval and consent to participate

Not applicable

## References

[1] Chapman J, Ismail AE, Dinu CZ. Industrial applications of enzymes: recent advances, techniques, and outlooks. Catalysts 2018;8. 10.3390/catal8060238.

[2] Chandra P, Enespa, Singh R, Arora PK. Microbial lipases and their industrial applications: a comprehensive review. Microb Cell Fact 2020;19. 10.1186/s12934-020-01428-8. 10.1186/s12934-020-01428-8 PMC7449042 32847584

[3] Jaeger KE, Reetz MT. Microbial lipases form versatile tools for biotechnology. Trends Biotechnol 1998;16. 10.1016/S0167-7799(98)01195-0. 10.1016/s0167-7799(98)01195-0 9744114

[4] Carranza LAS, Guerrero MMH, Gaibor MPA, Torres AFE, Vázquez AJS. Characterization, classification and uses of lipase enzymes in industrial production. Revista Cubana de Investigaciones Biomedicas 2020;39.

[5] Barus T, Suwanto A, Tri Wahyudi A, Wijaya H. Role of bacteria in tempe bitter taste formation: microbiological and molecular biological analysis based on 16S rRNA gene. Microbiol Indones 2008;2. 10.5454/mi.2.1.4.

[6] Hagedorn S, Kaphammer B. Microbial biocatalysis in the generation of flavor and fragrance chemicals. Annu Rev Microbiol 1994;48. 10.1146/annurev.mi.48.100194.004013. 10.1146/annurev.mi.48.100194.004013 7826026

[7] Nur Wulan, Sugeng Maryanto, Indri Mulyasari. The effect of fermentation time on protein and fat content in the red beans (Phaseolus Vulgaris L.) tempeh. JURNAL GIZI DAN KESEHATAN 2021;13. 10.35473/jgk.v13i2.236.

[8] Astuti M, Meliala A, Dalais FS, Wahlqvist ML. Tempe, a nutritious and healthy food from Indonesia. Asia Pac J Clin Nutr 2000;9. 10.1046/j.1440-6047.2000.00176.x. 10.1046/j.1440-6047.2000.00176.x 24394511

[9] Messaoudi A, Belguith H, Gram I, Hamida J Ben. Classification of EC 3.1.1.3 bacterial true lipases using phylogenetic analysis. Afr J Biotechnol 2010;9. 10.5897/ajb10.721.

[10] Reis P, Holmberg K, Watzke H, Leser ME, Miller R. Lipases at interfaces: a review. Adv Colloid Interface Sci 2009;147–148. 10.1016/j.cis.2008.06.001. 10.1016/j.cis.2008.06.001 18691682

[11] Ramani K, Chockalingam E, Sekaran G. Production of a novel extracellular acidic lipase from Pseudomonas gessardii using slaughterhouse waste as a substrate. J Ind Microbiol Biotechnol 2010;37. 10.1007/s10295-010-0700-2. 10.1007/s10295-010-0700-2 20204455

[12] Nisar S, Hanif MA, Rashid U, Hanif A, Akhtar MN, Ngamcharussrivichai C. Trends in widely used catalysts for fatty acid methyl esters (Fame) production: a review. Catalysts 2021;11. 10.3390/catal11091085.

[13] Mhetras NC, Bastawde KB, Gokhale D v. Purification and characterization of acidic lipase from Aspergillus niger NCIM 1207. Bioresour Technol 2009;100. 10.1016/j.biortech.2008.08.016. 10.1016/j.biortech.2008.08.016 18835775

[14] Nur N, Meryandini A, Suhartono MT, Suwanto A. Lipolytic bacteria and the dynamics of flavor production in Indonesian tempeh. Biodiversitas 2020;21. 10.13057/biodiv/d210850.

[15] Motohashi K. A novel series of high-efficiency vectors for TA cloning and blunt-end cloning of PCR products. Sci Rep 2019;9. 10.1038/s41598-019-42868-6. 10.1038/s41598-019-42868-6 PMC6478821 31015513

[16] Inoue H, Nojima H, Okayama H. High efficiency transformation of Escherichia coli with plasmids. Gene 1990;96. 10.1016/0378-1119(90)90336-P. 10.1016/0378-1119(90)90336-p 2265755

[17] Biasini M, Bienert S, Waterhouse A, Arnold K, Studer G, Schmidt T, et al. SWISS-MODEL: modelling protein tertiary and quaternary structure using evolutionary information. Nucleic Acids Res 2014;42. 10.1093/nar/gku340. 10.1093/nar/gku340 PMC4086089 24782522

[18] Gupta N, Rathi P, Gupta R. Simplified para-nitrophenyl palmitate assay for lipases and esterases. Anal Biochem 2002;311. 10.1016/S0003-2697(02)00379-2. 10.1016/s0003-2697(02)00379-2 12441161

[19] Laemmli UK. Cleavage of structural proteins during the assembly of the head of bacteriophage T4. Nature 1970;227. 10.1038/227680a0 5432063

[20] Demirbaş A. Biodiesel from vegetable oils via transesterification in supercritical methanol. Energy Convers Manag 2002;43. 10.1016/S0196-8904(01)00170-4.

[21] Masomian M, Jasni AS, Rahman RNZRA, Salleh AB, Basri M. Impact of signal peptide and transmembrane segments on expression and biochemical properties of a lipase from Bacillus sphaericus 205y. J Biotechnol 2017;264. 10.1016/j.jbiotec.2017.10.014. 10.1016/j.jbiotec.2017.10.014 29107669

[22] Sutcliffe IC, Russell RRB. Lipoproteins of gram-positive bacteria. J Bacteriol 1995;177. 10.1128/jb.177.5.1123-1128.1995. 10.1128/jb.177.5.1123-1128.1995 PMC176714 7868582

[23] Käll L, Krogh A, Sonnhammer ELL. Advantages of combined transmembrane topology and signal peptide prediction-the Phobius web server. Nucleic Acids Res 2007;35. 10.1093/nar/gkm256. 10.1093/nar/gkm256 PMC1933244 17483518

[24] Akoh CC, Lee GC, Liaw YC, Huang TH, Shaw JF. GDSL family of serine esterases/lipases. Prog Lipid Res 2004;43. 10.1016/j.plipres.2004.09.002. 10.1016/j.plipres.2004.09.002 15522763

[25] Brady L, Brzozowski AM, Derewenda ZS, Dodson E, Dodson G, Tolley S, et al. A serine protease triad forms the catalytic centre of a triacylglycerol lipase. Nature 1990;343. 10.1038/343767a0. 10.1038/343767a0 2304552

[26] Arpigny JL, Jaeger KE. Bacterial lipolytic enzymes: classification and properties. Biochem J 1999;343. 10.1042/0264-6021:3430177. PMC1220539 10493927

[27] Yuan D, Lan D, Xin R, Yang B, Wang Y. Biochemical properties of a new cold-active mono- and diacylglycerol lipase from marine member Janibacter sp. strain HTCC2649. Int J Mol Sci 2014;15. 10.3390/ijms150610554. 10.3390/ijms150610554 PMC4100168 24927145

[28] Angkawidjaja C, Kanaya S. Family I.3 lipase: bacterial lipases secreted by the type I secretion system. Cell Mol Life Sci. 2006;63. 10.1007/s00018-006-6172-x. 10.1007/s00018-006-6172-x PMC11136109 17103114

[29] Miskin JE, Farrell AM, Cunliffe WJ, Holland KT. Propionibacterium acnes, a resident of lipid-rich human skin, produces a 33 kDa extracellular lipase encoded by gehA. Microbiology (N Y) 1997;143. 10.1099/00221287-143-5-1745. 10.1099/00221287-143-5-1745 9168624

[30] Sommer P, Bormann C, Götz F. Genetic and biochemical characterization of a new extracellular lipase from Streptomyces cinnamomeus. Appl Environ Microbiol 1997;63. 10.1128/aem.63.9.3553-3560.1997. 10.1128/aem.63.9.3553-3560.1997 PMC168661 9293006

[31] Chahiniana H, Sarda L. Distinction between esterases and lipases: comparative biochemical properties of sequence-related carboxylesterases. Protein Pept Lett 2009;16. 10.2174/092986609789071333. 10.2174/092986609789071333 19508178

[32] Ozturkoglu-Budak S, Wiebenga A, Bron PA, de Vries RP. Protease and lipase activities of fungal and bacterial strains derived from an artisanal raw ewe’s milk cheese. Int J Food Microbiol 2016;237. 10.1016/j.ijfoodmicro.2016.08.007. 10.1016/j.ijfoodmicro.2016.08.007 27541978

[33] Lawrence RC, Fryer TF, Reiter B. The production and characterization of lipases from a micrococcus and a pseudomonad. J Gen Microbiol 1967;48. 10.1099/00221287-48-3-401. 10.1099/00221287-48-3-401 6052631

[34] Ayu E, Suwanto A, Barus T. Klebsiella pneumoniae from Indonesian tempeh were genetically different from that of pathogenic isolates. Microbiol Indones 2014;8. 10.5454/mi.8.1.2.

[35] Rosano GL, Ceccarelli EA. Recombinant protein expression in Escherichia coli: advances and challenges. Front Microbiol 2014;5. 10.3389/fmicb.2014.00172. 10.3389/fmicb.2014.00172 PMC4029002 24860555

[36] Neubauer P, Fahnert B, Lilie H, Villaverde A. Protein inclusion bodies in recombinant bacteria. Inclusions in Prokaryotes, 2006. 10.1007/7171_009.

[37] Ollis DL, Cheah E, Cygler M, Dijkstra B, Frolow F, Franken SM, et al. The a/b hydrolase fold. Protein Eng 1992;5. 10.1093/protein/5.3.197 1409539

[38] Ginalski K. Comparative modeling for protein structure prediction. Curr Opin Struct Biol 2006;16. 10.1016/j.sbi.2006.02.003. 10.1016/j.sbi.2006.02.003 16510277

[39] Schrag JD, Cygler M (1997). Lipases and alpha/beta hydrolase fold. Methods Enzymol.

[40] Babu DP, Bhakyaraj R, Vidhyalakshmi R. A low cost nutritious food “tempeh”-a review. vol. 4. 2009.

[41] Kamal Z, Yedavalli P, Deshmukh M v., Rao NM. Lipase in aqueous-polar organic solvents: activity, structure, and stability. Protein Sci. 2013;22. 10.1002/pro.2271. 10.1002/pro.2271 PMC3719085 23625694

[42] Plou FJ, Ferrer M, Nuero OM, Calvo M v., Alcalde M, Reyes F, et al. Analysis of Tween 80 as an esterase/lipase substrate for lipolytic activity assay. Biotechnol Techniques 1998;12. 10.1023/A:1008809105270.

[43] Sakai Y, Hayatsu M, Hayano K. Use of Tween 20 as a substrate for assay of lipase activity in soils. Soil Sci Plant Nutr 2002;48. 10.1080/00380768.2002.10409263.

[44] Mogensen JE, Sehgal P, Otzen DE. Activation, inhibition, and destabilization of Thermomyces lanuginosus lipase by detergents. Biochemistry 2005;44. 10.1021/bi0479757. 10.1021/bi0479757 15683256

[45] Katiyar M, Ali A. Effect of metal ions on the hydrolytic and transesterification activities of Candida rugosa Lipase. J Oleo Sci 2013;62. 10.5650/jos.62.919. 10.5650/jos.62.919 24200940

[46] Malvade AV, Satpute ST. Production of palm fatty acid distillate biodiesel and effects of its blends on performance of single cylinder diesel engine. Procedia Eng. 2013; 64. 10.1016/j.proeng.2013.09.230.

